# VDR Decrease Enhances the Efficacy of 1,25-Dihydroxyvitamin D3 Inhibiting Gefitinib Resistance by Regulating EGFR/FASN Loop in NSCLC Cells

**DOI:** 10.3390/ph18081238

**Published:** 2025-08-21

**Authors:** Junqing Yang, Mingyu Fang, Mengjun Hou, Yalei Duan, Jiali Wang, Kaiyong Hu, Shuo Liu, Xiaoying Liu, Xiaohan Peng, Xuansheng Ding, Zhirong Jia

**Affiliations:** 1 School of Basic Medicine and Clinical Pharmacy, China Pharmaceutical University, Nanjing 211198, China; 2 School of Pharmacy, China Pharmaceutical University, Nanjing 211198, China; 3 Precision Medicine Laboratory, School of Basic Medicine and Clinical Pharmacy, China Pharmaceutical University, Nanjing 211198, China

**Keywords:** VDR, 1,25(OH)_2_D_3_, FASN, NSCLC, gefitinib resistance

## Abstract

**Background**: Gefitinib is a first-generation epidermal growth factor receptor tyrosine kinase inhibitor (EGFR-TKI) targeting EGFR-mutated non-small cell lung cancer (NSCLC) and is a current first-line treatment for NSCLC. However, acquired resistance leads to the failure of treatment and remains a challenge. Therefore, identifying novel therapeutic approaches to combat EGFR-TKI resistance is crucial. **Methods**: The Cancer Genome Atlas (TCGA) database analysis and gefitinib-resistant cell lines were used to analyze VDR expression in NSCLC. Cell proliferation and apoptosis were assessed via MTT assay, colony formation assay, and flow cytometry. Immunofluorescence, qPCR, and Western blotting were used to measure mRNA and protein expression levels of VDR and other related molecules. Xenograft tumors in BALB/c nude mice were employed to investigate the effects of VDR and 1,25-dihydroxyvitamin D3 (1,25(OH)_2_D_3_) on gefitinib-resistant tumors in vivo. **Results**: We found that VDR was significantly upregulated in EGFR-TKI-resistant NSCLC cells. Patients with high VDR expression exhibited poor prognosis. VDR knockdown significantly inhibited cell proliferation, tumor growth, and reduced gefitinib resistance, whereas VDR overexpression enhanced resistance. VDR knockdown downregulated EGFR and FASN expression. Silencing either EGFR or FASN confirmed the existence of a positive feedback regulatory loop involving VDR, EGFR, and FASN. Treatment with 1,25(OH)_2_D_3_ increased VDR levels but decreased EGFR and FASN expression. VDR knockdown significantly enhanced the inhibitory effect of 1,25(OH)_2_D_3_ on gefitinib resistance. The combination of VDR knockdown and 1,25(OH)_2_D_3_ treatment was more effective than either treatment alone in suppressing EGFR and FASN expression. **Conclusions**: VDR promotes NSCLC resistance to EGFR-TKIs by regulating EGFR and FASN expression through a positive feedback loop. Knocking down VDR effectively enhances the ability of 1,25(OH)_2_D_3_ to overcome gefitinib resistance, mediated by the synergistic downregulation of EGFR and FASN expression. Targeting VDR represents a potential strategy to enhance the efficacy of 1,25(OH)_2_D_3_ in overcoming EGFR-TKI resistance.

## 1. Introduction

Globally, lung cancer remains the most frequent cancer and the primary cause of cancer mortality [1,2]. EGFR-TKIs (epidermal growth factor receptor tyrosine kinase inhibitors), targeted small-molecule inhibitors, have demonstrated the ability to extend survival in patients with EGFR-activating mutations [3]. Although the third-generation EGFR-TKIs can conquer resistance caused by the T790M mutation and are now the first-line standard for treating EGFR-mutant NSCLC (non-small cell lung cancer), acquired resistance remains a challenge [4]. Thus, identifying novel therapeutic means or agents for combating EGFR-TKI-resistant lung cancer is of critical importance.

Increased circulating vitamin D contents have been associated with reduced risks of various cancers [5], including lung cancer [6,7]. Both our studies [8,9] and those of others [10] have shown that the active metabolite of vitamin D3, 1,25(OH)_2_D_3_ (1,25-dihydroxyvitamin D3), mitigates EGFR-TKI resistance in NSCLC. 1,25-dihydroxyvitamin D3 modulates gene transcription by binding to the VDR (vitamin D receptor), which exerts transcriptional control by interacting with VDREs (vitamin D response elements) on target genes through its DNA-binding domains [11]. VDR plays a pivotal role in tumorigenesis and tumor development across multiple malignancies [12,13]. Studies confirm VDR overexpression in lung cancer and its association with poor prognosis in EGFR-mutated NSCLC patients [14,15]. However, its function and underlying mechanisms in NSCLC EGFR-TKI resistance remain unclear; furthermore, whether VDR is involved in the capacity of 1,25-dihydroxyvitamin D3 to counteract EGFR-TKI resistance in NSCLC warrants further investigation.

Targeting EGFR activation has proven to be an effective approach for overcoming EGFR-TKI resistance in NSCLC [16]. Reducing the expression of EGFR can effectively inhibit the resistance of NSCLC to EGFR tyrosine kinase inhibitors (EGFR-TKIs) by suppressing p-EGFR and the EGFR signaling pathway [17,18]. Previous investigations have demonstrated that 1,25(OH)_2_D_3_ reduces EGFR mRNA and protein levels in breast cancer cells [19], and downregulates EGFR expression and signaling in A431 cells [20]. Additionally, there exist VDRE in the promoters of EGFR, and VDR could bind to the EGFR VDRE sequence in the absence of ligands, indicating that VDR may regulate the transcription of EGFR [19,21], and EGFR-VDR crosstalk has been observed in colorectal cancer [22], suggesting a functional relationship between VDR and EGFR. These results indicate that 1,25(OH)_2_D_3_ and VDR have the potential to influence EGFR-TKI sensitivity by modulating EGFR signaling.

Emerging evidence suggests that the fatty acid metabolic pathway plays a crucial role in EGFR-TKI resistance [23,24]. De novo fatty acid synthesis is involved in EGFR activation [25,26]. The hepatic metabolic microenvironment can enhance de novo palmitate biosynthesis in liver metastatic CRC cells, promoting EGFR palmitoylation and thereby enhancing EGFR stability and plasma membrane localization [27]. Critically, FASN-mediated palmitoylation positively regulates EGFR-TKI-resistant EGFR-mutant NSCLC cells [28]. Notably, a positive feedback loop exists between EGFR and the synthesis of fatty acids. EGFR has been demonstrated to directly modulate fatty acid synthesis, thereby promoting lung cancer progression [29]. Moreover, FASN (fatty acid synthase), a pivotal rate-limiting enzyme in fatty acid biosynthesis, facilitates EGFR-TKI resistance by activating EGFR in the NSCLC cells. Inhibiting FASN has been found to impede EGFR activation and impair the growth of TKI-resistant EGFR-mutant NSCLC cells [28,30]. Thus, modulating the interaction between EGFR and FASN may represent a potential therapeutic approach for acquired EGFR-TKI resistance in NSCLC. However, the relationship between VDR, EGFR, and FASN remains to be determined.

Previous studies have suggested that AKT could be regulated by VDR and is a target of 1,25(OH)_2_D_3_ and VDR [31,32], and that VDR promotes the phosphorylation of the PI3K/AKT pathway in ovariectomy-induced osteoporosis of rats [33]. Moreover, the PI3K/AKT pathway is one of the downstream signaling cascades of EGFR [30]. EGFR activation can stimulate PI3K/Akt signaling, thereby enhancing the expression of SREBP-1 and increasing FASN synthesis, which promotes lipogenesis [34,35,36]. Concurrently, the AKT/mTOR pathway has also been demonstrated to facilitate de novo lipogenesis, and it exhibits a direct association with FASN [37,38,39,40]. In lung cancer, inhibition of the PI3K/AKT/mTOR signaling pathway downregulates FASN-mediated fatty acid synthesis [41]. These findings collectively indicate a connection between the EGFR–AKT pathway and FASN. In this study, the relationship between VDR, EGFR, FASN, and AKT was investigated.

Notably, 1,25-dihydroxyvitamin D3 and its analogs have been shown to inhibit FASN expression and alter fatty acid metabolism in prostate [42,43], ovarian [44], and colon [45] cancer cells. These findings led us to investigate whether 1,25(OH)_2_D_3_ could mitigate EGFR-TKI resistance by regulating EGFR and FASN. In this study, we explored the role of VDR and 1,25(OH)_2_D_3_ in modulating the interplay between EGFR and FASN, as well as the role and mechanisms by which VDR contributes to 1,25(OH)_2_D_3_-mediated inhibition of gefitinib resistance in NSCLC.

## 2. Results

### 2.1. Downregulation of VDR Expression Enhances the Gefitinib Cytotoxicity of NSCLC Cells

**Figure 1:**
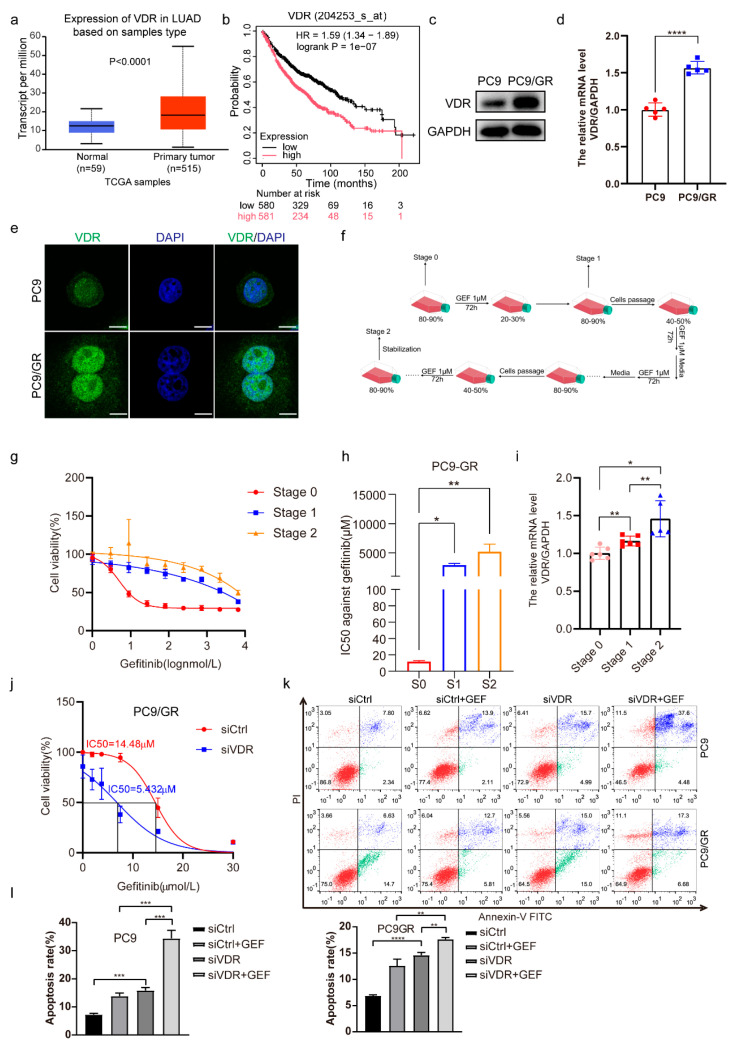
Downregulation of VDR expression enhances the gefitinib cytotoxicity of NSCLC cells. (**a**) VDR expression levels in LUAD patient tissues (*n* = 515) and normal lung tissues (*n* = 59) in TCGA were analyzed on UALCANC. (**b**) Kaplan–Meier analyses of survival probabilities of LUAD patients were performed on Kaplan–Meier Plotter. The log-rank test was used to calculate *p* values. (**c**) VDR protein levels in PC9 and PC9/GR cells were assessed by Western blotting (biological repeat = 3). (**d**) The mRNA levels of *VDR* were evaluated by QRT-PCR in PC9 and PC9/GR cells (mean ± SD; *n* = 5; **** *p* < 0.0001). (**e**) Immunofluorescence analysis of VDR from PC9 and PC9/GR cells. (Scale bar: 20 μm; original magnification: ×630; representative images from three experiments). (**f**) The steps of inducing gefitinib-resistant PC9 cells. (**g**–**i**) The cell viability (mean ± SD; *n* = 6), IC50 against gefitinib (mean ± SD; *n* = 3), and mRNA levels of *VDR* (mean ± SD; *n* = 5–6) were detected in cells of stage 0 (S0), stage 1 (S1), and stage 2 (S2). * *p* < 0.05, ** *p* < 0.01. (**j**) PC9/GR cells were transfected with siVDR or its negative control (siCtrl); after 24 h, the cells were treated with various concentrations of gefitinib (0, 1.875, 3.75, 7.5, 15, 30 μM) for another 48 h, then the cell cytotoxicity (mean ± SD; *n* = 5) and the IC50 value against gefitinib were calculated. (**k**,**l**) The PC9 and PC9/GR cells were transfected with siCtrl and siVDR for 6 h, then the cells were treated with gefitinib (5 μM) for another 48 h, and the apoptotic rates were assessed and analyzed by flow cytometry (mean ± SD; *n* = 3; ** *p* < 0.01, *** *p* < 0.001, **** *p* < 0.0001).

VDR expression in NSCLC was analyzed using data from TCGA (The Cancer Genome Atlas, https://www.cancer.gov/ccg/access-data, accessed on 14 September 2022) via UALCAN (University of Alabama at Birmingham Cancer Data Analysis Portal) [46]. We found that VDR expression was elevated in LUAD (lung adenocarcinoma) patients compared to normal lung tissues (Figure 1a). Furthermore, using median values as cutoffs, Kaplan–Meier relapse-free survival analysis exhibited an adverse correlation between VDR and LUAD patients’ survival [47] (Figure 1b). Next, we observed that both the protein and mRNA levels of VDR were significantly increased in PC9/GR (gefitinib-resistant) cells compared to PC9 cells (Figure 1c,d). Immunofluorescence analyses further confirmed that VDR predominantly localized in the nucleus with significantly higher nuclear VDR expression in PC9/GR cells compared with that in PC9 cells (Figure 1e). A rapid method for inducing gefitinib-resistant PC9 cells was established (Figure 1f), and cells at three stages (Stage 0–2) were collected. The results demonstrated a gradual increase in IC50 values against gefitinib with prolonged exposure (Figure 1g,h). Additionally, VDR transcript levels progressively increased with extended gefitinib exposure (Figure 1i). Moreover, cell viability and apoptosis assays proved that combining VDR knockdown with gefitinib treatment enhanced sensitivity to gefitinib compared to the single-treatment group in both PC9/GR and PC9 cells (Figure 1j–l). Collectively, we discovered that in gefitinib-resistant cells, VDR expression is upregulated, and that VDR knockdown may alleviate gefitinib resistance.

### 2.2. VDR Expression Correlates with EGFR and FASN

**Figure 2:**
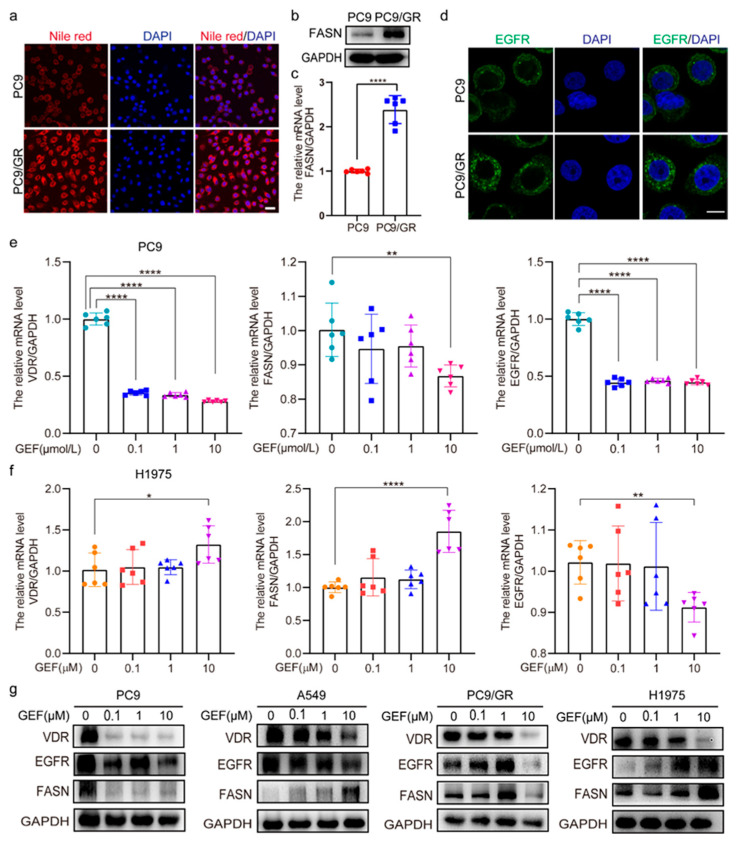
VDR expression correlates with EGFR and FASN. (**a**) Nile red staining was carried out to determine the lipid level in PC9 and PC9/GR cells. (Scale bar: 100 μm; original magnification: ×200; representative images from three experiments). (**b**,**c**) The expression of FASN in PC9 and PC9/GR cells was detected by Western blotting (biological repeat = 3) and QRT-PCR (mean ± SD; *n* = 6; **** *p* < 0.0001). (**d**) Immunofluorescence analysis of EGFR from PC9 and PC9/GR cells. (Scale bar: 20 μm; original magnification: ×630; representative images from three experiments). (**e**,**f**) PC9 and H1975 cells were treated with various concentrations of gefitinib (0.1, 1, 10 μM) for 6 h, then the mRNA levels of *VDR*, *FASN*, and *EGFR* were detected by QRT-PCR. (Mean ± SD; *n* = 6; * *p* < 0.05, ** *p* < 0.01, **** *p* < 0.0001). (**g**) PC9, A549, PC9/GR, and H1975 cells were treated with various concentrations of gefitinib (0.1, 1, 10 μM) for 48 h, then Western blotting was performed to evaluate the expression of VDR, EGFR, and FASN.

Compared to PC9 cells, lipid levels, protein, and mRNA levels of FASN were significantly elevated in PC9/GR cells (Figure 2a–c). Moreover, significantly increased EGFR expression was also observed in PC9/GR cells (Figure 2d). To investigate the relationship between VDR, EGFR, and FASN, gefitinib was used to treat the NSCLC cells. The results showed that gefitinib dramatically reduced the mRNA and protein levels of VDR, EGFR, and FASN in PC9 cells (Figure 2e–g). However, in H1975 cells treated with low concentrations (0.1 μM and 1 μM) of gefitinib, no significant alterations in VDR, EGFR, or FASN mRNA levels were detected (Figure 2f). Similarly, when A549, H1975, and PC9/GR cells were exposed to low concentrations (0.1 μM and 1 μM) of gefitinib, the protein levels of VDR, EGFR, and FASN exhibited only a modest change (Figure 2g). These findings suggest a correlation between VDR expression and EGFR/FASN in NSCLC cells.

### 2.3. The Interaction Among VDR, EGFR, FASN, and AKT Regulates Gefitinib Sensitivity

**Figure 3:**
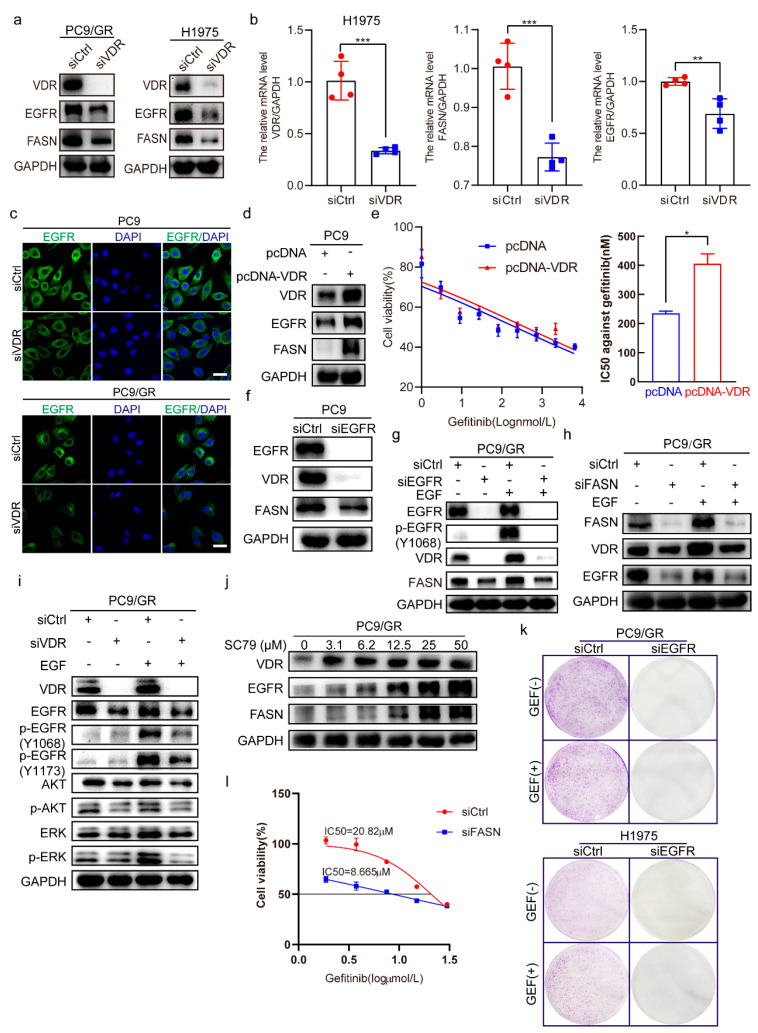
The interaction among VDR, EGFR, and FASN regulates gefitinib sensitivity. (**a**) PC9/GR and H1975 cells were transfected with siCtrl or siVDR. After 48 h, Western blotting was performed to detect the expression of VDR, EGFR, and FASN. (**b**) H1975 cells were transfected with siCtrl or siVDR for 48 h, then the mRNA levels of *VDR*, *FASN*, and *EGFR* were detected. (Mean ± SD; *n* = 4; ** *p* < 0.01, *** *p* < 0.001). (**c**) PC9 and PC9/GR cells were transfected with siCtrl or siVDR for 48 h, then the immunofluorescence assay was carried out to detect the EGFR expression. (Scale bar: 50 μm; original magnification: ×400; representative images from three experiments). (**d**) PC9 cells were transfected with pcDNA3.1-VDR or empty vector for 48 h, then the expression of VDR, EGFR, and FASN was detected by Western blotting. (**e**) PC9 cells were transfected with pcDNA3.1-VDR or empty vector; after 6 h, the cells were treated with various concentrations of gefitinib (0, 1, 3, 9, 27, 81, 243, 729, 2187, 6561 nM) for another 48 h, then the cell viability (mean ± SD; *n* = 6) and IC50 against gefitinib were calculated. (Mean ± SD; *n* = 3; * *p* < 0.05). (**f**) PC9 cells were transfected with siEGFR or siCtrl for 48 h, then the protein level of EGFR, VDR, and FASN was determined by Western blotting. (**g**–**i**) PC9/GR cells were transfected with siEGFR, siFASN, siVDR, or siCtrl for 48 h, then the cells were stimulated with or without EGF (50 ng/mL) for 15 min, and the expression of indicated proteins was detected by Western blotting. (**j**) PC9/GR cells were treated with various concentrations of SC79 for 48 h, then Western blotting was performed to evaluate the expression of VDR, EGFR, and FASN. (**k**) PC9/GR and H1975 cells were transfected with siEGFR or siCtrl; after 24 h, cells were treated with or without gefitinib (1 μM). Treatments were repeated every 3 days. Colony formation was assessed by crystal violet staining. (**l**) PC9/GR cells were transfected with siFASN or siCtrl; after 24 h, the cells were treated with various concentrations of gefitinib (0, 1.875, 3.75, 7.5, 15, 30 μM) for another 48 h, then the cell viability (mean ± SD; *n* = 5) and the IC50 value against gefitinib were calculated.

In PC9/GR and H1975 cells, VDR knockdown via small interfering RNA (siRNA) reduced EGFR and FASN protein levels (Figure 3a). Moreover, downregulated *EGFR* and *FASN* mRNA levels in H1975 cells (Figure 3b). In PC9 and PC9/GR cells, immunofluorescence assay further confirmed that VDR knockdown decreased EGFR expression (Figure 3c). Conversely, VDR overexpressed in PC9 cells via pcDNA3.1-VDR plasmid transfection significantly upregulated EGFR and FASN expression (Figure 3d) and reduced gefitinib cytotoxicity (Figure 3e). To further investigate the interplay among VDR, EGFR, and FASN in PC9 and PC9/GR cells, individual knockdowns of EGFR, VDR, and FASN were conducted with or without EGF. The results indicated that EGFR knockdown in PC9 cells significantly reduced VDR and FASN expression, with a pronounced decrease in VDR (Figure 3f). In PC9/GR cells, similar results were observed regardless of EGF treatment (Figure 3g). Moreover, FASN knockdown in PC9/GR cells markedly reduced EGFR and VDR expression independent of EGF treatment (Figure 3h). VDR knockdown in PC9/GR cells inhibits EGF-induced EGFR signaling activation (Figure 3i). Additionally, SC79, an AKT activator, induced dose-dependent increases in VDR, EGFR, and FASN expression (Figure 3j). In PC9/GR cells and H1975 cells, Reducing EGFR expression significantly inhibited the colony formation with or without gefitinib (Figure 3k). Cell viability assays indicated that the combined FASN knockdown and gefitinib treatment increased gefitinib sensitivity more than single treatments (Figure 3l). Taken together, these findings suggest a positive feedback loop among VDR, EGFR, and FASN in NSCLC cells, and disrupting this loop may alleviate NSCLC EGFR-TKI resistance.

### 2.4. 1,25(OH)_2_D_3_ Increases VDR Expression and Decreases Expression of EGFR and FASN

**Figure 4:**
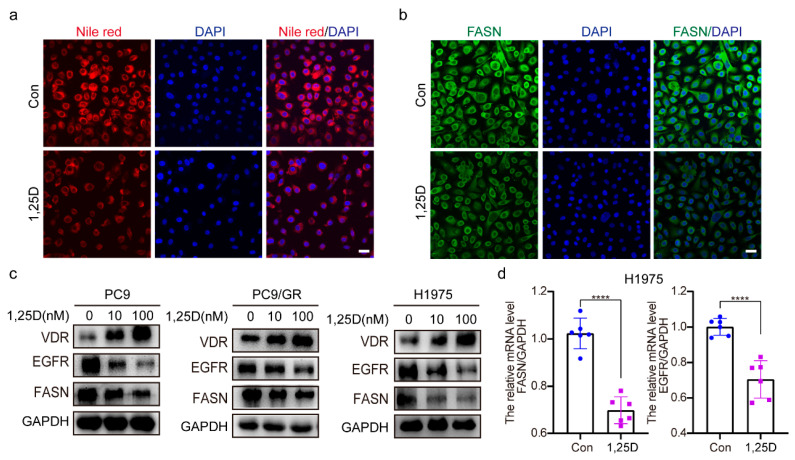
1,25(OH)_2_D_3_ increases VDR expression and decreases expression of EGFR and FASN. (**a**,**b**) PC9/GR cells were treated with 1,25(OH)_2_D_3_ (1,25D) (100 nM) for 48 h, then the lipid level and FASN expression were detected by Nile red staining and immunofluorescence, respectively. (Scale bar: 100 μm; original magnification: ×200; representative images from three experiments). (**c**) PC9, PC9/GR, and H1975 cells were treated with 1,25D (10 nM and 100 nM) for 48 h, then the expression of VDR, EGFR, and FASN was detected by Western blotting. (**d**) H1975 cells were treated with 1,25D (100 nM) for 6 h, then the mRNA levels of FASN and EGFR were determined by QRT-PCR (mean ± SD; *n* = 6; **** *p* < 0.0001).

Next, we investigated the impact of 1,25(OH)_2_D_3_ (1,25D) on lipid levels and the expression of VDR, EGFR, and FASN. The results indicate that treatment with 1,25(OH)_2_D_3_ significantly reduces lipid accumulation (Figure 4a) and FASN levels (Figure 4b) in PC9/GR cells. Additionally, 1,25(OH)_2_D_3_ increases VDR protein expression while decreasing EGFR and FASN protein expression in H1975, PC9, and PC9/GR cells (Figure 4c). 1,25(OH)_2_D_3_ also downregulated EGFR and FASN mRNA levels in H1975 cells (Figure 4d). The research findings indicate that 1,25(OH)_2_D_3_ upregulates VDR while suppressing EGFR and FASN expression.

### 2.5. Knockdown of VDR Combined with 1,25(OH)_2_D_3_ Increases Gefitinib Cytotoxicity by Inhibiting EGFR and FASN in NSCLC Cells

**Figure 5:**
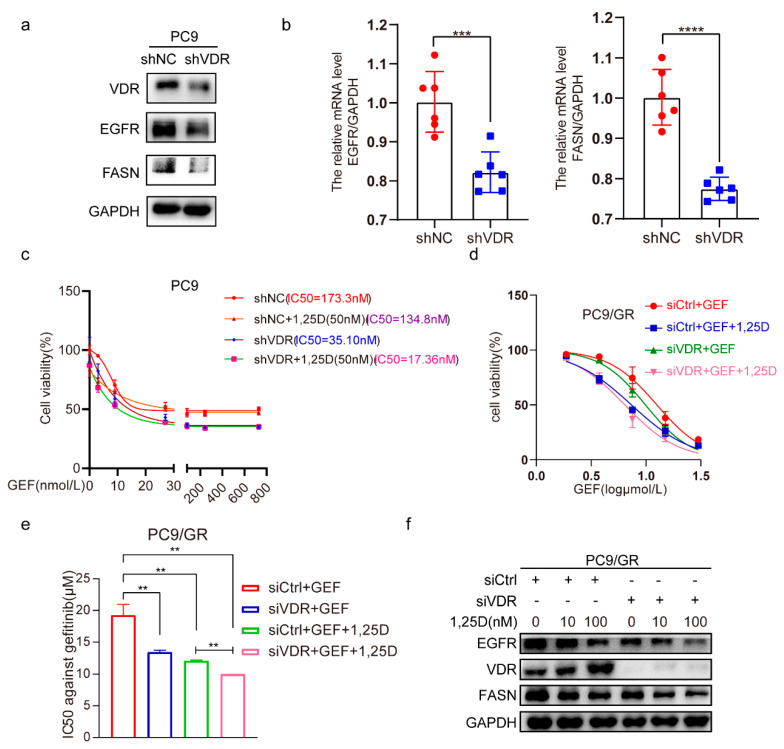
Knockdown of VDR combined with 1,25(OH)_2_D_3_ increases gefitinib cytotoxicity by inhibiting EGFR and FASN in NSCLC cells. (**a**,**b**) The PC9 cells were infected with LV-shVDR or LV-shNC, then the expression of VDR, EGFR, and FASN was investigated. (Mean ± SD; *n* = 6; *** *p* < 0.001, **** *p* < 0.0001). (**c**) The PC9 cells were infected with LV-shVDR or LV-shNC, and were treated with 1,25D (50 nM) and gefitinib (0, 3, 9, 27, 81, 243, 729 nM) for 48 h, then the cell viability (mean ± SD; *n* = 6) and IC50 against gefitinib were calculated. (**d**,**e**) PC9/GR cells were transfected with siVDR or siCtrl, and were treated with 1,25D (50nM) and gefitinib (0, 1.875, 3.75, 7.5, 15, 30 μM) for 48 h, then the cell viability (mean ± SD; *n* = 5) and IC50 against gefitinib were calculated (mean ± SD; *n* = 3; ** *p* < 0.01). (**f**) PC9/GR cells were transfected with siVDR or siCtrl, and were treated with 1,25D (10 nM, 100 nM) and gefitinib (1 μM) for 48 h, then the expression of EGFR, VDR, and FASN was detected.

By infecting PC9 cells with lentivirus-shRNA-VDR, we observed that VDR knockdown strongly reduced the protein expression levels of EGFR and FASN (Figure 5a), and similarly downregulated their mRNA levels (Figure 5b). Cell viability assays revealed that the cells treated with the combination of VDR knockdown and 1,25(OH)_2_D_3_ were more sensitive to gefitinib compared to those receiving either treatment alone (Figure 5c). Consistently, similar results were observed in PC9 cells transfected with siVDR, showing reduced EGFR and FASN expression (Figure 5d,e). Notably, the combination of VDR silencing and 1,25(OH)_2_D_3_ treatment further suppressed EGFR and FASN expression levels compared to either treatment alone (Figure 5f). The above findings show that targeting VDR combined with 1,25(OH)_2_D_3_ effectively enhances gefitinib cytotoxicity by downregulating EGFR and FASN in NSCLC cells.

### 2.6. Knockdown of VDR Combined with 1,25(OH)_2_D_3_ Inhibits Tumor Growth by Suppressing EGFR and FASN In Vivo

**Figure 6:**
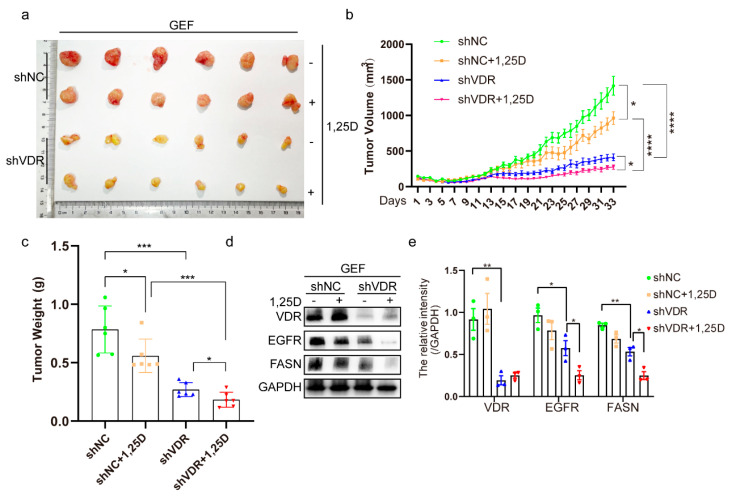
Knockdown of VDR combined with 1,25(OH)_2_D_3_ inhibits tumor growth by suppressing EGFR and FASN in vivo. (**a**) Macroscopic appearance of PC9/GR xenografts of each group. (**b**,**c**) Tumor sizes and weight were presented as mean ± SD (*n* = 6); * *p* < 0.05, *** *p* < 0.001, **** *p* < 0.0001. (**d**,**e**) Whole protein cell lysates were prepared randomly from 3 tumors per group for Western blot to detect the indicated proteins (mean ± SD; *n* = 3; * *p* < 0.05, ** *p* < 0.01).

A xenograft mouse model was created in vivo via subcutaneously injecting shNC- or shVDR-transfected cells. The combination of shVDR and gefitinib markedly inhibited tumor progression in comparison to shNC and gefitinib. Notably, 1,25(OH)_2_D_3_ further suppressed tumor growth when combined with shVDR and gefitinib compared to shVDR and gefitinib alone (Figure 6a–c). Additionally, VDR downregulation reduced EGFR and FASN expression, and the combination of VDR knockdown and 1,25(OH)_2_D_3_ further diminished EGFR and FASN levels compared to shVDR alone (Figure 6d,e). These findings demonstrate that VDR downregulation enhances the efficacy of 1,25(OH)_2_D_3_ to mitigate gefitinib resistance.

## 3. Discussion

In this research, we explored the association between the VDR and gefitinib resistance in NSCLC cells, focusing on the function of VDR in 1,25(OH)_2_D_3_-mediated regulation of gefitinib resistance, which has not been previously reported. Our findings indicate that reducing VDR expression inhibits gefitinib resistance in NSCLC by disrupting the EGFR–FASN interplay. Additionally, 1,25(OH)_2_D_3_ enhances VDR expression while downregulating EGFR and FASN. Notably, combining VDR inhibition with 1,25(OH)_2_D_3_ further suppresses EGFR and FASN expression, effectively counteracting gefitinib resistance. Thus, VDR downregulation enhances the efficiency of 1,25(OH)_2_D_3_ in decreasing gefitinib resistance through modulation of the EGFR/FASN loop.

Initially, we observed that VDR expression was significantly elevated in PC9/GR cells compared with gefitinib-sensitive PC9 cells. Moreover, VDR expression progressively increased as PC9 cells were exposed to gefitinib. AKT activation was found to upregulate VDR expression. However, the precise mechanisms underlying VDR upregulation and its expression across various EGFR-TKI-resistant cell lines require further investigation. Knocking down VDR in PC9/GR cells reduced cell viability and tumor growth, with the combination of VDR silencing and gefitinib treatment exhibiting a more pronounced effect than either intervention alone. Interestingly, VDR downregulation also enhanced the efficacy of 1,25(OH)_2_D_3_ to reduce gefitinib resistance in both in vivo and in vitro models. These findings highlight that inhibiting VDR expression enhances gefitinib cytotoxicity and strengthens the efficacy of 1,25(OH)_2_D_3_ in mitigating gefitinib resistance.

From a clinical perspective, VDR is widely expressed in various tumor tissues and plays a role in cancer development, although its expression varies among cancer types. VDR is upregulated in all gynecological malignancies [13], whereas reduced expression has been observed in colorectal cancer [48]. However, the relationship between VDR with NSCLC remains unclear. TCGA database analysis indicated that VDR levels were increased in LUAD patients in comparison with normal lung tissues. Furthermore, Kaplan–Meier relapse-free survival analysis demonstrated a reverse relationship between VDR levels and relapse-free survival among LUAD patients. Consistent with these findings, our study confirmed that VDR levels are elevated in gefitinib-resistant NSCLC cells and that VDR silencing mitigates gefitinib resistance both in vitro and in vivo.

A deeper understanding of VDR function is crucial for future research. We demonstrated that VDR regulates EGFR/FASN crosstalk, establishing a positive feedback loop among VDR, EGFR, and FASN in NSCLC cells. Disrupting this loop may offer a therapeutic approach to overcome EGFR-TKI resistance. EGFR inhibitors have been widely used to overcome EGFR-TKI resistance [16], while FASN, a key factor in cancer cell survival, has been linked to poor prognosis, recurrence risk, and drug resistance [34]. Blocking EGFR–FASN interactions via VDR inhibition may therefore provide a novel therapeutic avenue for treating acquired NSCLC EGFR-TKI resistance.

A particularly intriguing aspect of our study is the efficacy of 1,25(OH)_2_D_3_ to upregulate VDR expression [49], aligning with our observations. This led us to investigate the function of VDR in 1,25(OH)_2_D_3_-mediated inhibition of NSCLC EGFR-TKI resistance. Our results confirmed that VDR silencing enhances the efficacy of 1,25(OH)_2_D_3_ in suppressing gefitinib resistance, and combining VDR knockdown with 1,25(OH)_2_D_3_ intervention further inhibits EGFR and FASN expression compared to a single treatment. Evidence shows that 1,25(OH)_2_D_3_ controls its own activity through negative feedback signaling. It rapidly induces the binding of the VDR/RXR (retinoic X receptor) heterodimer to the promoter sequence of 24-hydroxylase cytochrome P450 family 24 subfamily A member 1 (CYP24A1) [50], leading to CYP24A1 upregulation and a subsequent decrease in 1,25(OH)_2_D_3_ levels [51]. Acquired gefitinib-resistant NSCLCs upregulate their own VDR protein expression, which may induce CYP24A1 production and accelerate the catabolism of 1,25(OH)_2_D_3_, thereby suppressing the anti-tumor effects of 1,25(OH)_2_D_3_. Previous studies have reported that VDR knockout (VDR−/−) mice exhibit significantly reduced metastatic lung cancer growth, likely due to increased serum concentrations of 1,25(OH)_2_D_3_ [52]. Studies have shown that 1,25(OH)_2_D_3_ can inhibit HCC growth by disrupting the activation of the downstream translational machinery of the PI3K/AKT pathway via a VDR-independent mechanism [53]. Additionally, independent of VDR, 1,25(OH)2D3 has been demonstrated to induce acid sphingomyelinase in gastric cancer cells [54] and activate autolysosomal degradation against Helicobacter pylori via the PDIA3 receptor [55]. Moreover, vitamin D3 metabolites can suppress the expression of lipogenic genes via inhibiting the function of sterol regulatory element-binding protein (SREBP), a key transcription factor of lipid synthesis, via interaction with SREBP cleavage-activating protein (SCAP), independent of canonical VDR function [56]. According to the above, the suppression of gefitinib resistance and EGFR/FASN expression mediated by 1,25(OH)_2_D_3_ may occur through VDR-independent mechanisms. The development of a VDR-silent vitamin D [56] derivative could represent a promising strategy for overcoming EGFR-TKI resistance in NSCLC.

In conclusion, our research elucidates the role of VDR downregulation in enhancing gefitinib cytotoxicity and disrupting the EGFR/FASN crosstalk. Furthermore, we demonstrate that VDR silencing potentiates the efficacy of 1,25(OH)_2_D_3_ in suppressing gefitinib resistance by downregulating EGFR and FASN expression. To our knowledge, this is the first study to present the role and mechanism of VDR in NSCLC EGFR-TKI resistance, offering a novel approach to enhance the potential value of 1,25(OH)_2_D_3_ in NSCLC through targeting VDR.

## 4. Materials and Methods

### 4.1. Cell Lines

NSCLC lines PC9 and PC9/GR (kind gifts from Shanghai Pulmonary Hospital; EGFR exon 19 deletion, no harboring T790M mutation and MET gene amplification), H1975 (National Collection of Authenticated Cell Cultures, Shanghai, China; EGFR L858R/T790M mutation), and A549 (a kind gift from 3D Medicines, Shanghai, China; wild-type EGFR) were cultured in DMEM (Dulbecco modified Eagle medium) (KeyGEN BioTECH, Nanjing, China) and supplemented with 10% FBS (ExCell Bio, Shanghai, China) in a humidified cell incubator at 37 °C with an atmosphere of 5% CO_2_.

### 4.2. Reagents and Chemicals

Primers were obtained from Genscript (Nanjing, China). 1,25(OH)_2_D_3_ (calcitriol) and gefitinib were ordered from Selleck (Shanghai, China). SC79 was purchased from Beyotime Biotechnology (Nantong, China). EGF was from PeproTech (Rocky Hill, NJ, USA).

### 4.3. Western Blot Analysis

Total proteins were extracted from NSCLC cells or tumor tissues using the lysis buffer (KeyGEN BioTECH, Jiangsu, China) containing a mixture of protease inhibitors (KeyGEN, Jiangsu, China), and the proteins were quantified and used for Western blotting analysis. The supernatant of the extracted protein lysate was denatured and separated by SDS-polyacrylamide gel electrophoresis, and proteins of different molecular weights were transferred to PVDF (polyvinylidene difluoride membranes, Millipore, MA, USA) by membrane transfer. Following an overnight incubation at 4 °C with specific primary antibodies, the membranes were probed with the indicated antibodies: VDR and p-EGFR (Y1086) (Cell Signaling Technology, Boston, MA, USA); EGFR and AKT1/2/3 (Bimake, Houston, TX, USA); FASN, ERK1/2, and PhosphoERK1/2 (Thr202/Tyr204) (p-ERK) (Proteintech Group, WUHAN SANYING, WuHan, China); Phospho-pan-AKT1/2/3 (Ser473) (p-AKT) and p-EGFR (Y1173) (Affinity, Changzhou, China); GAPDH (Proteintech Group, Wuhan, China). The membrane was washed three times and then incubated with goat anti-rabbit IgG secondary antibody with HRP label (Beyotime, Shanghai, China) for 1 h at room temperature on a shaker at slow speed. According to the manufacturer’s instructions, protein detections were carried out using an electrochemiluminescence substrate (Vazyme, Nanjing, China) with visualization on a Tanon imaging system (Tanon, Shanghai, China). Protein expression was evaluated by ChemiScope (Clinx, Shanghai, China).

### 4.4. Quantitative Real-Time PCR

Using Trizol reagent (Vazyme, Nanjing, China), the total RNA was extracted from NSCLC cells prior to cDNA synthesis (Vazyme). GAPDH mRNA levels served as an internal control for normalization. The primer sequences utilized for real-time PCR were human VDR: 5′-CAGTTCGTGTGAATGATGG-3′ (forward, F), 5′-TGAAGAGGTGATACAGTGAT-3′ (reverse, R); human EGFR: 5′-TGGATGATAGACGCAGATAG-3′ (F), 5′-CACGGTAGAAGTTGGAGT-3′ (R); human FASN: 5′-TATGCGACGGGAAAGTAT-3′ (F), 5′-TGTGGAT GATGCTGATGA-3′ (R); human GAPDH: 5′-C TTCTTTTGCGTCGCCAGCCGA-3′ (F), 5′-ACCAGGCGCCCAATACGACCAA-3′ (R). The real-time PCR was performed using Applied Biosystems (Foster City, CA, USA), and relative gene expressions were analyzed using the ∆∆Ct method.

### 4.5. Cell Viability Assays

The method of cell viability was described previously [57]. Briefly, 5 × 10^3^ cells per well were seeded in 96-well plates. After 16–24 h, the NSCLC cells were transfected with the specified siRNA, plasmid, or exposed to designated treatments for 48 h. Absorbance was examined at the indicated time.

### 4.6. Colony-Formation Assay

The cells were first transfected with siCtrl or siEGFR, and then treated with gefitinib (2 μM) after 24 h. Then, after 14 days of incubation and colony formation, the medium was discarded and the cells were fixed with methanol and stained with 0.1% crystal violet (KeyGEN BioTECH, Jiangsu, China).

### 4.7. siRNA and Plasmid Transfection

For all transfections, 150 pmol siRNA (the target sequence of VDR-specific siRNA: 5′-AGUUCAUUCUGACAGAUGA-3′, EGFR-specific siRNA: 5′-GCAACAUGU CGAUGGACUU-3′, FASN-specific siRNA: 5′-GGGACAGUGCAUCAAAGAA-3′) (Genomeditech, Shanghai, China) or 1 μg of pcDNA3.1-VDR or empty vector (Corues Biotechnology, Nanjing, China) was transfected into 70–80% confluent cells. Lipofectamine 2000 reagent (Life Technologies, Carlsbad, CA, USA) was used for transfections of siRNA or plasmid into PC9/GR, H1975, or PC9 cells growing in DMEM (high glucose, serum-free, KeyGEN BioTECH, Jiangsu, China). After 4–6 h, the serum-free media was switched out for DMEM with 10% FBS. The following tests were conducted at specified time points post-transfection.

### 4.8. Flow Cytometry for the Detection of Apoptosis

Cell apoptosis detection was assessed using an Annexin V-FITC apoptosis kit (Vazyme, Nanjing, China) per the manufacturer’s instructions. Cells underwent transfection with siCtrl or siVDR for 6 h; following a 48-h gefitinib exposure, cells were collected for detecting the apoptosis rate, and the total apoptosis rate was detected by FACS flow cytometry (Miltenyi Biotec, Bergisch Gladbach, Germany). Then, the data were processed using the FlowJo VX program.

### 4.9. The Steps of Inducing Gefitinib-Resistant PC9 Cells

PC9 cells were cultured with gefitinib (1 μM) for 72 h to eliminate most parental cells. The surviving cells (about 20–30%) were raised in gefitinib-free medium until reaching 80–90% confluence, marking the first stage of drug-resistant cells (Stage 1). These stage 1 cells (40–50%) were subsequently treated with 1 μM gefitinib for another 72 h and then maintained in a gefitinib-free medium. This process was repeated until the cell density recovered to 80–90%. The cells (40–50%) were then continuously cultured in a medium containing 1 μM gefitinib, and the medium was replaced with fresh medium every 72 h until the cell density reached 80–90% again. The cells were then passaged and maintained in gefitinib-containing medium for an additional two weeks, at which point the second stage of drug-resistant cells was defined (stage 2).

### 4.10. Immunofluorescence

In PC9 and PC9/GR cells, immunofluorescence was used to detect VDR (Cell Signaling Technology, Danvers, MA, USA), EGFR (Bimake, Houston, TX, USA), and FASN (Proteintech Group, Wuhan, China) expression. DAPI (Beyotime, Shanghai, China) was used for nuclear counterstaining, and a laser scanning confocal microscope (Carl Zeiss, Jena, Germany) was employed for visualization under identical imaging conditions.

### 4.11. Nile Red Staining

The method of Nile red staining referred to a previous study [58]. In short, the cells were cultured on cover glasses for 24 h, then the cells were fixed in 4% PFA (paraformaldehyde, Biosharp, Beijing, China) for 20 min at room temperature, and were incubated with Nile red (1:2000, MCE, Shanghai, China) in PBS for 10 min. Counterstaining with DAPI was performed before imaging on a confocal microscope (Carl Zeiss, Oberkochen, Germany). A representative image is displayed based on three independent experiments.

### 4.12. Lentivirus Infection

PC9/GR or PC9 cells (1.5 × 10^5^) were inoculated in 6-well plates. After 16–24 h, the cells were subjected to stimulation with polybreen (2 μg/mL) and subsequently infected with lentivirus (1.07 × 10^8^ TU/mL) containing shNC or shVDR (contract identification number: GM-LC-125364, Genomeditech). After 72 h, cells were collected, and ZsGreen-positive cells were assessed using a Laser scanning confocal microscope (CLSM, Carl Zeiss LSM800) (*n* = 2, biological replicates); Western blot and QRT-PCR evaluated VDR, EGFR, and FASN expression (*n* = 6, biological replicates).

### 4.13. In Vivo Mouse Model

BALB/c nude male mice (18–20 g), aged 4 weeks, were ordered from Yangzhou University (Yangzhou, China). All experimental procedures involving animals received approval from the Ethics Committee of China Pharmaceutical University (approval number: 2025-01-051; approval date: 23 January 2025). The mice were housed in individually ventilated cages under a standard environment maintained at 24 ± 2 °C, with 55 ± 3% humidity, under a 12 h light/dark cycle from 8 a.m. to 8 p.m. and received free access to water and food. A xenograft mouse model was created as previously mentioned [59]. Briefly, the PC9/GR cells were infected using a lentivirus containing control shRNA (shNC) or shVDR (Genomeditech, Shanghai, China). Cells were digested and made into a cell resuspension with PBS after 3 days. The shNC and shVDR groups were randomly assigned into four groups of six mice each, and the cell suspension containing approximately 1.5 × 10^7^ cells was injected subcutaneously into the right forelimb of each mouse. When the average subcutaneous tumor volume reached 70–100 mm^3^, gefitinib (50 mg/kg/d), 1,25(OH)_2_D_3_ (1 μg/kg/2d), or solvent control alone was administered, respectively. Subcutaneous tumor volume was measured every 2 days and computed using the formula (length × width^2^)/2. Once the average tumor volume in the blank control group reached 1500 mm^3^, each mouse was euthanized, and tumor tissue samples were collected to measure the tumor mass. No blinding was performed for the animal experiments.

### 4.14. TCGA and Kaplan–Meier Analysis

VDR expression in NSCLC was analyzed using data from The Cancer Genome Atlas (TCGA) database (https://www.cancer.gov/ccg/access-data, accessed on 14 September 2022) via UALCAN (https://ualcan.path.uab.edu/, accessed on 14 September 2022). Kaplan–Meier survival analyses for LUAD patients based on VDR expression were conducted using Kaplan–Meier Plotter (https://kmplot.com/analysis/index.php?p=background, 21 September 2022).

### 4.15. Statistical Analysis

The data are expressed as the means ± standard deviation. Two-sided statistical tests were conducted. Student’s *t*-test compared two groups, while one-way ANOVA with Dunnett’s test assessed multiple groups. Statistical analysis was performed using Prism 8.00 software (GraphPad, San Diego, CA, USA). Two-tailed *p*-values < 0.05 were considered significant.

## Data Availability

The data that support the findings of this study are available from the corresponding author upon reasonable request.

## Abbreviations

The following abbreviations are used in this manuscript:

EGFR	epidermal growth factor receptor
EGFR-TKIs	epidermal growth factor receptor tyrosine kinase inhibitors
NSCLC	non-small cell lung cancer
TCGA	The Cancer Genome Atlas
1,25(OH)_2_D_3_	1,25-dihydroxyvitamin D3
VDR	vitamin D receptor
VDREs	vitamin D response elements
FASN	fatty acid synthase
LUAD	lung adenocarcinoma
PC9/GR	PC9/gefitinib-resistant strain (gefitinib-resistant PC9)
SREBP	sterol regulatory element-binding protein
SCAP	SREBP cleavage-activating protein
IC50	half maximal inhibitory concentration
